# 

**Published:** 1867-06

**Authors:** 


### CHICAGO

### MEDICAL JOURNAL.

VOL. XXIV. JUNE, 1867. NO. 6.

CHICAGO:

GEORGE H. FERGUS, BOOK AND JOB PRINTER,

12 & 14 CLARK STREET.

